# Associations of maternal early-pregnancy dietary glycemic index with childhood general, abdominal and ectopic fat accumulation

**DOI:** 10.1016/j.clnu.2021.02.046

**Published:** 2021-03-06

**Authors:** Rama J. Wahab, Vincent W.V. Jaddoe, Romy Gaillard

**Affiliations:** a The Generation R Study Group, Erasmus MC, University Medical Center, Rotterdam, The Netherlands; b Department of Pediatrics, Sophia’s Children’s Hospital, Erasmus MC, University Medical Center, Rotterdam, The Netherlands

**Keywords:** Childhood obesity, Pregnancy, Glycemic index, Diet

## Abstract

**Background & aims:**

Maternal hyperglycemia during pregnancy is an important risk factor for childhood adiposity. Maternal dietary glycemic index during pregnancy directly influences maternal and fetal glucose concentrations. We examined the associations of maternal early-pregnancy dietary glycemic index with offspring general, abdominal and ectopic fat accumulation among normal weight and overweight or obese pregnant women and their offspring.

**Methods:**

In a population-based cohort study among 2488 Dutch pregnant women and their children, we assessed maternal dietary glycemic index by food frequency questionnaire at median 13.4 (95% range 10.7; 21.1) weeks gestation. Dietary glycemic index was used continuously and categorized into low (≤55), normal (56–69) and high (≥70) glycemic index diet. We measured offspring BMI, total fat mass and android/gynoid fat mass ratio by DXA, and visceral fat mass and liver fat fraction by MRI at 10 years.

**Results:**

No associations of maternal early-pregnancy dietary glycemic index with offspring adiposity were present among normal weight women and their children. Among overweight and obese women and their children, 1-Standard Deviation Score (SDS) increase in maternal early-pregnancy dietary glycemic index was associated with higher childhood BMI (0.10 SDS, 95% Confidence Interval (CI) 0.01; 0.19), total fat mass index (0.13 SDS, 95% CI 0.05; 0.22), visceral fat mass index (0.19 SDS, 95% CI 0.07; 0.32) and tended to be associated with a higher android/gynoid fat mass ratio (0.09 SDS, 95% CI −0.01; 0.19) and higher risk of childhood overweight (Odds Ratio (OR) 1.20, 95% CI 0.97; 1.48). Overweight and obese women consuming an early-pregnancy low-glycemic index diet, as compared to an early-pregnancy normal-glycemic index diet, had children with lower BMI, total fat mass index, visceral fat mass index and android/gynoid fat mass ratio at 10 years (p-values<0.05). No women consumed a high-glycemic index diet. No associations were explained by maternal socio-economic, lifestyle and dietary characteristics, birth or childhood characteristics. No associations with liver fat fraction were present.

**Conclusions:**

In overweight or obese women and their children, a higher maternal early-pregnancy dietary glycemic index is associated with childhood general, abdominal and visceral fat accumulation, but not with liver fat. Intervention studies among overweight and obese pregnant women may need to target the dietary glycemic index to prevent childhood adiposity.

## 1 Introduction

Childhood overweight and obesity are major public health problems and associated with cardio-metabolic morbidity and premature mortality [1,2]. Childhood BMI is most commonly used as a measure to assess the risk of adverse cardio-metabolic health outcomes. However, ectopic fat accumulation, especially visceral fat and liver fat accumulation, are even more closely related to the development of an impaired glucose metabolism, low-grade systemic inflammation and dyslipidemia and subsequent cardio-metabolic diseases [3,4].

Accumulating evidence suggests that fetal life is a critical period for establishing the risk of obesity and ectopic fat accumulation in later life [5,6]. Maternal nutrition is an important factor for fetal development as it directly influences fetal nutrient supply [7]. The maternal dietary glycemic index and load influence maternal postprandial glucose concentrations, which is a main nutritional determinant of fetal growth, adipocyte development and metabolism [5,6,8]. A maternal diet with a high glycemic index and load may increase glucose transfer to the fetus, adversely affecting fetal growth and body composition [9,10] These effects may be even stronger among overweight or obese pregnant women, who are more likely to have an unhealthy diet and a suboptimal glucose metabolism [5]. Animal studies have already shown that a maternal low-glycemic index diet during pregnancy reduces offspring weight and visceral and liver fat accumulation, but associations among humans are not known [11,12]. An observational study among 906 pregnant women and their offspring showed that a higher maternal dietary glycemic index during early-pregnancy, but not during late-pregnancy, was associated with a higher childhood total body fat mass [13].

We hypothesized that a higher maternal early-pregnancy dietary glycemic index and load, especially among overweight or obese women, may be modifiable risk factors for the development of obesity and ectopic fat accumulation in the offspring. Therefore, in a population-based prospective cohort study among 2488 pregnant women and their children, we examined the associations of maternal early-pregnancy dietary glycemic index and load with childhood BMI, total fat mass, android/gynoid fat mass ratio, visceral fat mass, liver fat and the risk of overweight at 10 years. We examined these associations among normal weight and overweight or obese women and their offspring separately.

## 2 Methods

### 2.1 Study design and study sample

This study was embedded in the Generation R study, a population based prospective birth cohort study in Rotterdam, the Netherlands [14]. Written informed consent was obtained from all women. The study was approved by the local Medical Ethical Committee (MEC 198.782/2001/31). In total, 4096 Dutch women were enrolled during pregnancy of which 3558 women had information on dietary intake available. We excluded women with pregestational diabetes (n = 7) and non-singleton live births (n = 80). Of the remaining 3471 women and their offspring, 2488 participated in follow up measurements at 10 years (Fig. S1). Due to later implementation of MRI scans within follow-up visits, we only had measurements of visceral fat or liver fat fraction available within a subgroup of 1397 children.

### 2.2 Maternal dietary glycemic index and load

We obtained information on maternal dietary intake during early-pregnancy at a median of 13.4 weeks gestation (95% range 9.9; 22.6) by a semi-quantitative 293-item Food Frequency Questionnaire (FFQ) [14]. The FFQ was validated against three 24-h dietary recalls and biomarkers from blood samples in 80 pregnant women with Dutch ethnicity living in Rotterdam, the Netherlands, which is directly comparable to our study population. Energy-adjusted intra-class correlation coefficients for macronutrient intakes ranged from 0.41 to 0.88 and was 0.60 for carbohydrate intake [15]. The average energy intake and carbohydrate intake was calculated using the Dutch Food Composition Table 2006 [16]. To calculate maternal early-pregnancy dietary glycemic index and load, we assigned glycemic index values to each individual food item in the FFQ. We obtained glycemic index values from the glycemic index database on the Dutch diet published by the Medical Research Council Human Nutrition Research (MRC HNR), Cambridge, United Kingdom, using glucose as reference (glycemic index for glucose equal to 100). We calculated the mean glycemic index and load per day [17]. We considered the dietary glycemic index as our main exposure, as the dietary glycemic index provides information on the quality of the glycemic response to a carbohydrate containing food product and is more often used in intervention studies and clinical settings [9,18]. We included dietary glycemic load as a secondary exposure, as this measure takes the amount of carbohydrate intake into account and therefore provides additional information on maternal postprandial glucose concentrations, but this measure may be more prone to measurement error [19–21]. We constructed standard deviation scores to analyze maternal early-pregnancy dietary glycemic index and glycemic load across the full range.

In line with previous intervention studies, we also categorized the mean maternal early-pregnancy dietary glycemic index per day into a low-, normal-, and high-glycemic index diet, using similar cut-offs as used for individual food products (low-glycemic index diet (≤55), a normal-glycemic index diet (56–69) and a high-glycemic index diet (≥70)) [22,23].

### 2.3 Childhood general, abdominal and ectopic fat

At the age of 10 years, we measured height and weight without shoes and heavy clothing and calculated Body Mass Index (BMI) (kg/m^2^). Childhood BMI sex-and-age-adjusted standard (SDS) were constructed based on Dutch reference growth charts (Growth Analyzer 4.0, Dutch Growth Research Foundation) [24]. We defined childhood overweight or obesity by categorizing childhood weight status according to the International Obesity Task Force cut-offs [25]. Overweight and obesity were combined into one category and under- and normal weight were combined into one category.

We measured total, android and gynoid body fat mass by Dual-Energy X-ray absorptiometry (DXA) (iDXA; General Electrics–Lunar, 2008, Madison, WI) and calculated android/gynoid fat mass ratio [26]. Visceral fat and liver fat fraction were obtained from MRI scans as described previously [27]. Childhood body fat mass is strongly influenced by height of the child [28]. To enable assessment of the associations of maternal early-pregnancy dietary glycemic index and load with childhood adiposity outcomes independent of childhood size, we estimated the optimal adjustment for childhood height using log–log regressions (details in Supplementary Methods 1) [28,29]. We calculated total fat mass index (total fat mass/height^4^) and visceral fat mass index (visceral fat mass/height^3^).

### 2.4 Covariates

Information on maternal age, educational level, parity, folic acid supplement use, prepregnancy weight and diagnosis of pre-gestational diabetes were obtained through questionnaire at enrolment. Information on smoking, alcohol consumption, vomiting and maximum weight during pregnancy was obtained through questionnaires throughout pregnancy. Information on maternal dietary energy, fiber, fat and protein intake during pregnancy was obtained with the FFQ. As a proxy measurement of maternal overall diet quality, the Dietary Approaches to Stop Hypertension (DASH) diet score was derived from the FFQ [30–32]. The score is composed of 8 food components, based mainly on the Fung method with a scoring system based on quintile rankings and included the intake of total grains, vegetables, fruits, non-full-fat dairy products, and nuts/seeds/legumes and the intake of red and processed meats, sugar-sweetened beverages/sweets/added sugars and sodium. A lower maternal DASH diet score characterizes a lower overall dietary quality [32]. We measured maternal height at enrolment and calculated prepregnancy body mass index (BMI) [14]. Information on gestational diabetes, child’s gestational age and weight at birth and sex were obtained from medical records. Information on breastfeeding and the timing of introduction to solid foods was assessed by questionnaires during infancy, and the average time watching television was assessed by a questionnaire at 10 years of age [14].

### 2.5 Statistical analyses

First, we performed a non-response analysis to compare characteristics of women with and without offspring follow-up measurements available. Second, we examined the associations of maternal early-pregnancy dietary glycemic index SDS and maternal dietary glycemic index categories with childhood BMI, total fat mass index, android/gynoid fat mass ratio, visceral fat mass index, liver fat fraction and the risk of obesity using linear and logistic regression models. We constructed five different models, based on a Directed Acyclic Graph (DAG) analysis to identify which factors may act as confounders or potential mediators in these associations (Supplementary Methods S2): 1) the basic model, adjusted for gestational age at intake, fetal sex and child’s age at follow up visit; 2) the confounder model (main model), the basic model additionally adjusted for potential confounders. We only included maternal age, parity, educational level, prepregnancy BMI, smoking during pregnancy, vomiting during pregnancy, and daily total energy intake during pregnancy to the confounder model based on their association with the exposure and outcome of interest and a >10% change of the effect estimate after adding these covariates to the basic model [33–35]. Based on these criteria, alcohol consumption and folic acid supplementation were not selected for inclusion in the confounder model.; 3) the birth model, the confounder model additionally adjusted for gestational-age-and-sex-adjusted birth weight; 4) the child model, the birth model additionally adjusted for infant breastfeeding, solid food introduction and television watching at 10 years; 5) the maternal diet model, the confounder model additionally adjusted for gestational weight gain and maternal fiber, protein and fat intake during pregnancy to assess the effects of other important maternal dietary factors strongly related to the glycemic index and overall diet quality on the observed associations. Based on our hypothesis that effects may be stronger among overweight and obese women, we performed all analyses in the total population and stratified for mothers with a prepregnancy BMI<25 kg/m^2^ and BMI≥25 kg/m^2^. We also formally tested for interactions of maternal prepregnancy BMI continuously with maternal early-pregnancy dietary glycemic index continuously for all childhood outcomes. We added the interaction term of maternal prepregnancy BMI with maternal early-pregnancy dietary glycemic index to linear regression models including maternal prepregnancy BMI, early-pregnancy dietary glycemic index and gestational age at intake, fetal sex, and child’s age at follow up visit. Significant interactions of maternal prepregnancy BMI with maternal early-pregnancy dietary glycemic index were present for childhood total fat mas index (p = 0.00) and childhood visceral fat mass index (p = 0.02), but not for childhood BMI (p = 0.20), childhood android/gynoid fat mass ratio (p = 0.17) and childhood liver fat (p = 0.86). We performed four sensitivity analyses to assess the robustness of our findings: 1) we repeated the analyses using maternal early-pregnancy dietary glycemic index in quartiles to further explore whether associations were stronger for women consuming a higher dietary glycemic index within our study population; 2) we repeated the analyses using maternal early-pregnancy dietary glycemic load as an exposure to assess the potential additional effect of quantity of maternal dietary carbohydrate intake; 3) we repeated the analyses additionally adjusting for the maternal DASH diet score to assess the potential effect of overall maternal diet quality on the associations; 4) we repeated the analyses excluding mothers with gestational diabetes, as we were interested in the effects of maternal early-pregnancy dietary glycemic index among a non-diabetic population. To reduce selection bias due to missing data, multiple imputations of covariates (pooled results of 5 imputed datasets) were performed [36]. The analyses were performed using the Statistical Package of Social Sciences version 24.0 for Windows (SPSS Inc., Chicago, IL, USA).

## 3 Results

### 3.1 Subject characteristics

Table 1 shows that the overall mean maternal early-pregnancy dietary glycemic index was 57.6 (SD 3.3) and was comparable for normal weight and overweight or obese women. 20.9% women within our population consumed a low-glycemic index diet, 79.1% women consumed a normal-glycemic index diet and no women consumed a high-glycemic index diet based on comparison to individual food products classifications. Non-response analyses showed that women with and without offspring participating in follow-up measurements had a similar early-pregnancy dietary glycemic index and prepregnancy BMI (Supplementary Table S1).

### 3.2 Maternal early-pregnancy dietary glycemic index and childhood general, abdominal and ectopic fat accumulation

Table 2 shows that among the total population and among normal weight women and their children, maternal early-pregnancy dietary glycemic index was not associated with childhood BMI, total body fat or ectopic fat accumulation. Among overweight or obese women and their children, a higher maternal early-pregnancy dietary glycemic index was associated with a higher childhood BMI, total fat mass index and visceral fat mass index and tended to be associated with a higher childhood android/gynoid fat mass ratio in the confounder model (differences: 0.10 SDS (95% confidence interval (CI) 0.01; 0.18), 0.13 SDS (95% CI 0.05; 0.22), 0.19 SDS (95% CI 0.07; 0.32) and 0.09 SDS (95% CI −0.01; 0.19) per SDS increase in maternal dietary glycemic index, respectively). Additional adjustment for gestational-age-and-sex-adjusted birth weight, child characteristics and additional maternal dietary characteristics did not explain these associations. No associations with liver fat fraction were present. Among overweight or obese women, a higher maternal early-pregnancy dietary glycemic index also tended to be associated with a higher risk of childhood overweight, but the association was not significant (Odds Ratio (OR) 1.20, 95% CI 0.97; 1.48 per SDS increase in maternal dietary glycemic index) (Table 3). Maternal early-pregnancy dietary glycemic index was not associated with the risk of childhood overweight in the total population or among normal weight women.

**Fig. 1:**
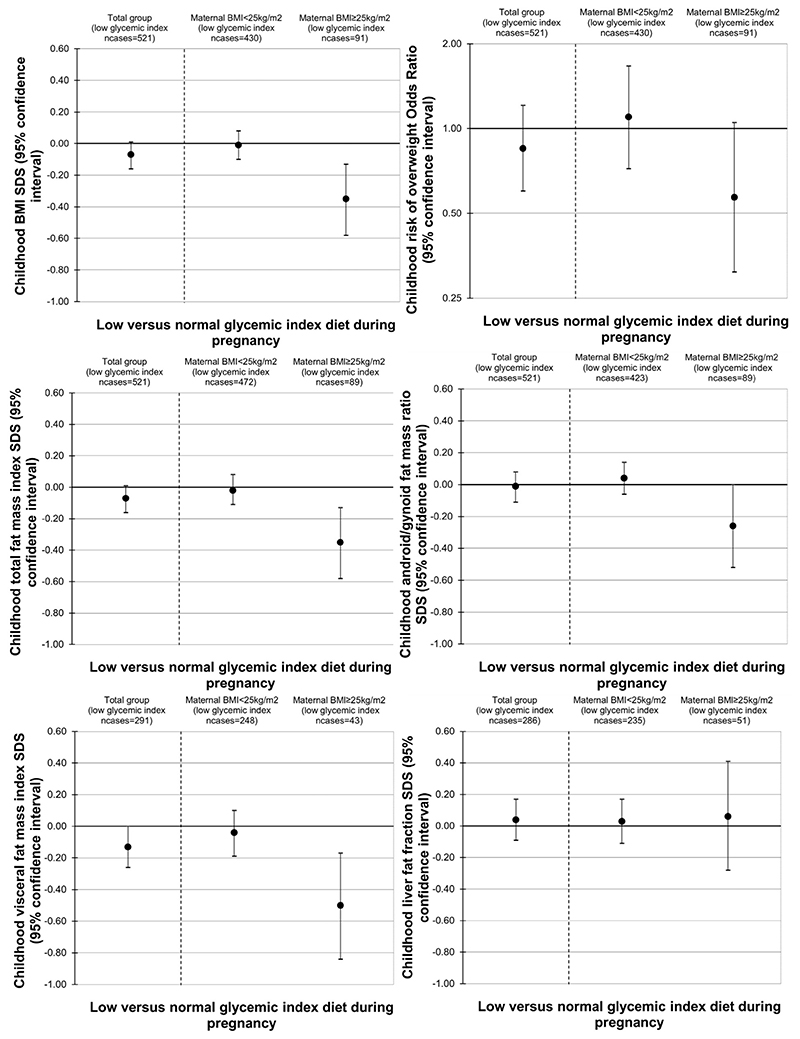
Associations of maternal early-pregnancy low-glycemic index diet as compared to maternal early-pregnancy normal-glycemic index diet with childhood adiposity outcomes. Cut offs for a low-, and normal-glycemic index diet are based on comparison to individual food products classifications (≤55 and 56–69 for a low-, and normal-glycemic index diet, respectively). No women within our population consumed a high-glycemic index diet (≥70). Ncases represents the number of women who consumed a low-glycemic index diet within the specified group. Values represent regression coefficients and odds ratios (ORs) (95% Confidence Intervals) from linear and logistic regression models respectively, that reflect differences in standard deviation scores for childhood adiposity outcomes and differences in risk for childhood overweight for a maternal low-glycemic index diet as compared to a normal-glycemic index diet. Associations were adjusted for maternal age, maternal educational level, maternal prepregnancy BMI, smoking during pregnancy, vomiting during early-pregnancy and daily total energy intake.

Figure 1 shows the associations of maternal dietary glycemic index categorized into low and normal-glycemic index diet according to individual food products classification with childhood general, abdominal and ectopic fat. Among the total population and among normal weighted women and their children, a maternal low-glycemic index diet, as compared to a normal-glycemic index diet, was not associated with childhood BMI, total body fat or ectopic fat. Among overweight or obese women and their children, a maternal low-glycemic index diet during pregnancy, as compared to a maternal normal-glycemic index diet, was associated with a lower childhood BMI (−0.35 SDS, 95% CI −0.58; −0.13), total fat mass index (−0.35 SDS, 95% CI 0.58; 0.13), android/gynoid fat mass ratio (−0.26 SDS, 95% CI −0.52; 0.00), visceral fat mass index (−0.50 SDS, 95% CI −0.84; −0.17) and tended to be associated with a lower risk of childhood overweight (OR 0.57, 95% CI 0.31; 1,05). No associations with liver fat fraction were present.

### 3.3 Sensitivity analyses

When repeating analyses using the maternal early-pregnancy dietary glycemic index in quartiles, associations of the highest quartile with childhood general, abdominal and ectopic fat accumulation as compared to the lowest quartile, were similar as for the analyses using the maternal early-pregnancy dietary glycemic continuously (Supplementary Table S2). Among the total population, a higher maternal early-pregnancy glycemic load was associated with a higher child total fat mass, whereas among normal weight women and their children no associations were present. Among overweight and obese women, a higher maternal early-pregnancy dietary glycemic load was associated with a higher childhood BMI, total fat mass index, visceral fat mass index, and higher risk of childhood overweight in the confounder models (Supplementary Tables S3 and S4). After adjustment for the maternal DASH diet score, effects estimates were similar to those observed after adjustment for maternal dietary characteristics (Supplementary Table S5). Excluding women with gestational diabetes from the analyses did not change our findings (Supplementary Table S6).

## 4 Discussion

Among overweight or obese women and their children, a higher maternal early-pregnancy dietary glycemic index was associated with higher childhood BMI, total body fat, abdominal fat and visceral fat accumulation, but not with liver fat accumulation. These associations were not explained by maternal socio-demographic, lifestyle and other dietary characteristics, birth or child characteristics. In normal weight women, no associations of maternal early-pregnancy dietary glycemic index with childhood general, abdominal or ectopic fat accumulation were present.

### 4.1 Interpretation of main findings

The maternal dietary glycemic index during pregnancy is receiving increasing interest as a potential modifiable target to improve birth outcomes and reduce the risk of offspring obesity [37]. The dietary glycemic index during pregnancy directly influences maternal postprandial glucose concentrations. Post-prandial peaks in maternal glucose concentrations and subsequent peak increases in fetal glucose and insulin concentrations could irreversibly affect offspring adiposity development by altering fetal growth, development of adipocytes and metabolism [6,8]. Intervention studies have already shown that stimulating a low-glycemic index diet during pregnancy in women at increased risk of an impaired glucose metabolism may reduce the risk of macro-somia and adiposity in infant offspring [38,39]. The long-term effects of a higher maternal dietary glycemic index during pregnancy on offspring adiposity and ectopic fat accumulation are not well-known. A study among 842 Irish mother–child pairs observed no association of maternal dietary glycemic index assessed between 12 and 16 weeks gestation with offspring BMI or waist circumference at 5 years after adjustment for maternal prepregnancy BMI [40]. The mean dietary glycemic index was 58.9 within this study population. Contrarily, a study among 906 mother–child pairs in the United Kingdom reported that a higher maternal dietary glycemic index and load in early-pregnancy, but not late-pregnancy, were associated with a higher offspring total body fat measured by DXA at 4 and 6 years of age after adjustment for maternal prepregnancy BMI. This study reported a mean dietary glycemic index of 59.6 in early-pregnancy and 58.9 in late-pregnancy. No differences in associations among normal weight and overweight or obese women and their offspring were present [13]. Animal studies showed that a maternal high-glycemic index diet during pregnancy was associated with higher offspring visceral fat mass and a transient higher liver fat, which normalized in adolescence [11,41].

Partly in line with these previous studies, we observed that among overweight and obese women and their children, a higher maternal early-pregnancy dietary glycemic index across the full range was associated with a higher childhood BMI and total body fat. In addition, we showed that a higher maternal early-pregnancy dietary glycemic index was associated with higher childhood abdominal and visceral fat accumulation. In line with previous human studies, we observed that women within our study consumed a diet with a relatively low mean dietary glycemic index of 57.7. Associations for maternal dietary glycemic load were in similar direction, which suggests that the observed associations of maternal early-pregnancy dietary glycemic index with childhood adiposity are not fully explained by the amount of carbohydrate intake. The associations for maternal dietary glycemic load were slightly weaker, possibly due to more measurement error and confounding in assessment of the dietary glycemic load than dietary glycemic index or a small effect of the amount of carbohydrate intake [42]. As compared to a maternal normal-glycemic index diet, a maternal low-glycemic index diet was associated with lower childhood BMI, total fat mass, android/gynoid fat mass and visceral fat accumulation. We observed no associations with childhood liver fat accumulation, what could be explained by our relatively healthy population, but also, as suggested by an animal study, the effects on liver fat fraction may be more pronounced in infancy [11]. Associations were only present among women with pre-pregnancy overweight or obesity and their children. Additional adjustment for prepregnancy BMI did not affect the observed associations within this group of women and their offspring. This is in line with intervention studies mainly reporting beneficial effects of a low-glycemic index diet during pregnancy on pregnancy outcomes among women at risk of an impaired glucose metabolism [22]. Overall, our findings suggest that among women with prepregnancy overweight or obesity and their children, a higher maternal early-pregnancy dietary glycemic index is associated with higher childhood higher general, abdominal and visceral fat accumulation at 10 years, but not with liver fat accumulation. No associations are present among normal weight women and their offspring.

The mechanisms underlying the observed associations are not well known. Our findings were not explained by maternal socio-economic, lifestyle, birth or childhood characteristics. Additional adjustment for other maternal macronutrients did not explain the associations, nor did additional adjustment for overall dietary quality by additionally adjusting for the maternal DASH diet score [43]. This suggests that observed associations are less likely to only reflect effects of an overall unhealthy lifestyle and diet among overweight or obese women, but that a higher maternal early-pregnancy dietary glycemic index may have a direct effect on offspring adipose tissue development. A maternal diet during pregnancy with a higher glycemic index and subsequent peaks in postprandial glucose concentrations increase glucose transfer to the developing embryo or fetus. Overweight or obese women have a more pronounced insulin resistance during pregnancy, causing larger fluctuations in postprandial glucose concentrations and higher glucose transfer to the developing embryo or fetus [5,6,44]. Higher embryonic and fetal glucose concentrations accelerate embryonic and fetal growth and may alter development of adipocytes, pancreatic endocrine and hepatic metabolic function [6,8,45]. These alterations may not only directly increase fetal adipose tissue accumulation, but could also cause fat accumulation in postnatal life due to irreversible changes in fatty acid oxidation, lipogenesis, and lipoprotein export [46,47]. Experimental studies need to identify mechanisms underlying the associations of maternal early-pregnancy dietary glycemic index on offspring adiposity development.

The observed associations of maternal early-pregnancy dietary glycemic index with childhood adiposity outcomes among overweight and obese women and their children were relatively small, but important from a public health perspective. It is well-known that childhood adiposity tracks into adulthood [48–52]. We observed the strongest effect of the maternal early-pregnancy dietary glycemic index with childhood visceral fat accumulation. Visceral fat accumulation is known to cause systemic inflammation and is strongly linked to an impaired glucose metabolism and an adverse lipid profile [48]. In adulthood, visceral fat accumulation strongly increases the risk of type 2 diabetes, cardiovascular disease and premature mortality, even irrespective of general fat mass [3,52]. Maternal prepregnancy overweight and obesity are associated with increased offspring risks of obesity and ectopic fat accumulation [3,4,6]. Especially in this high risk group, insight into modifiable lifestyle factors from preconception onwards is needed to develop interventions to improve offspring adiposity outcomes and related cardio-metabolic health. Intervention studies among overweight and obese women should reveal whether stimulating a low-glycemic index diet already from preconception or early-pregnancy reduces the risk of childhood obesity and increased general, abdominal and visceral fat in the offspring.

## 5 Methodological considerations

Strengths of this study are the prospective design, large sample size, and the use of detailed measures of childhood adiposity obtained with DXA and MRI. 72% of children from Dutch mothers with early-pregnancy dietary glycemic index available participated in follow-up measurements. As we observed no differences in early-pregnancy dietary glycemic index and prepregnancy BMI between mothers with and without offspring participating in follow up measurements, we consider bias due to loss the follow-up unlikely. The selection towards a relatively healthy Dutch population with a relatively low mean dietary glycemic index may affect the generalizability of our findings. Further studies are needed to replicate our findings among multi-ethnic populations with a more diverse dietary intake. Even though the FFQ is a validated questionnaire widely used for dietary assessment in observational studies, measurement of food intake by a FFQ may be affected by over- or underreporting of dietary intake during pregnancy. Although the mean dietary glycemic index is in line with previous studies, this limitation of the FFQ may explain the relatively low mean dietary glycemic index within our study population and lead to an underestimation of results [13,40]. However, a study performed within a study population directly comparable to our study population validate the FFQ using three 24-h dietary recalls and biomarkers from blood samples and showed only a slight underestimation of carbohydrate intake. Calculation of the dietary glycemic index from the FFQ may further be affected by uncertainty induced by preparation of foods, mixed dishes, variations of food products over time or unavailability of specific food products [17]. Further studies using different methods to assess the dietary glycemic index in low and high-risk pregnant populations are needed to replicate our results. We adjusted our analyses for multiple confounding factors, but residual confounding may still be present.

## 6 Conclusions

Among overweight and obese women and their children, a higher maternal early-pregnancy dietary glycemic index was associated with a higher childhood BMI, general and abdominal fat accumulation and visceral fat accumulation, but not with liver fat accumulation. Intervention studies among overweight and obese pregnant women may need to target the dietary glycemic index to prevent childhood adiposity.

## Supplementary Material

Supplementary data to this article can be found online at https://doi.org/10.1016/j.clnu.2021.02.046.

Supplementary data

## Figures and Tables

**Table 1:** Population characteristics according to maternal prepregnancy BMI.

	Total group (n = 2488)	Women with BMI<25 kg/m^2^ (n = 1681)	Women with BMI≥25 kg/m^2^ (n = 480)
**Maternal characteristics**			
Maternal age at enrolment, mean (SD), years	31.8 (4.1)	31.9 (4.2)	31.5 (4.0)
Gestational age at enrolment, median (95% range), weeks	13.4 (9.9; 22.6)	13.4 (9.9; 22.5)	13.3 (10.1; 23.2)
Parity, n nulliparous (%)	1549 (62.3)	1071 (63.8)	288 (60.0)
Pre-pregnancy BMI, median (95% range)	22.3 (18.4; 33.3)	21.6 (18.1; 24.7)	27.5 (25.1; 38.1)
Gestational weight gain, mean (SD), kg/week	0.35 (0.14)	0.36 (0.13)	0.33 (0.18)
Education, n high (%)	1566 (62.9)	1128 (67.9)	234 (49.0)
Dietary glycemic index, mean (SD)	57.7 (3.5)	57.6 (3.3)	57.9 (3.1)
First quartile, mean (SD)	53.7 (1.4)	53.7 (1.4)	53.8 (1.3)
Second quartile, mean (SD)	56.4 (0.6)	56.4 (0.6)	56.4 (0.6)
Third quartile, mean (SD)	58.5 (0.7)	58.5 (0.7)	58.5 (0.7)
Fourth quartile, mean (SD)	62.0 (1.9)	62.0 (1.8)	61.9 (1.9)
Dietary glycemic load, mean (SD)	154.0 (46.1)	154.7 (45.7)	151.1 (47.6)
Low glycemic index diet, n yes (%)	522 (20.9)	376 (22.4)	80 (16.5)
Carbohydrate intake, mean (SD), g/d	266 (74)	268 (73)	260 (76)
Protein intake, mean (SD), g/d	79 (19)	80 (19)	78 (18)
Fat intake, mean (SD), g/d	87 (24)	87 (24)	84 (23)
Fiber intake, mean (SD), g/d	24 (7)	24 (7)	22 (6)
Total energy intake, mean (SD), kcal/d	2145 (500)	2160 (499)	2090 (498)
Dietary Approaches to Stop Hypertension Diet Score, mean (SD)	25 (4)	25 (5)	24 (4)
Folic acid supplement use, n yes (%)	1865 (75.0)	1319 (91.7)	372 (90.1)
Alcohol use during pregnancy, n yes (%)	1577 (63.4)	1141 (71.4)	277 (60.1)
Smoking during pregnancy, n yes (%)	510 (20.5)	366 (22.8)	96 (20.9)
Vomiting during early-pregnancy, n yes (%)	93 (3.7)	55 (3.5)	27 (5.9)
Gestational diabetes, n yes (%)	20 (0.8)	9 (0.6)	10 (2.2)
**Birth/infant characteristics**			
Sex, n female (%)	1255 (50.4)	838 (49.9)	252 (52.5)
Gestational age at birth, median (95% range), weeks	40.3 (36.0; 42.4)	40.3 (36.3; 42.4)	40.3 (35.7; 42.4)
Birthweight, mean (SD), g	3498 (537)	3490 (528)	3560 (535)
Ever breastfed, n yes (%)	2006 (80.6)	1390 (93.3)	352 (87.8)
Introduction of solid foods before 6 months, n yes (%)	1633 (65.6)	1098 (86.7)	302 (90.7)
**Child characteristics at 10 years**			
Age, median (95% range), years	9.7 (9.4; 10.2)	9.7 (9.3; 10.4)	9.7 (9.3; 10.7)
Average television watching time >2 h/day, n yes (%)	511 (20.5)	308 (18.3)	137 (28.3)
BMI, median (95% range), kg/m^2^	16.6 (14.3; 21.3)	16.4 (14.3; 20.3)	17.5914.6; (23.8)
Total fat mass, median (95% range), g	8038 (4463; 18,644)	7718 (4421; 17,191)	9710 (4792; 22,408)
Android/gynoid fat mass ratio, median (95% range)	0.23 (0.15; 0.45)	0.23 (0.15; 0.40)	0.26 (0.16; 0.50)
Visceral fat mass, median (95% range), g	372 (166; 928)	353 (157; 820)	457 (197; 1198)
Liver fat fraction, median (95% range), %	2.0 (1.2; 4.6)	1.9 (1.3; 3.5)	2.2 (1.4; 4.8)
Overweight or obese, n yes (%)	285 (11.4)	129 (7.7)	120 (25.0)

**Table 2:** Associations of maternal early-pregnancy dietary glycemic index with childhood general, abdominal and ectopic fat accumulation.

	Effect estimates for childhood outcome per SDS increase in maternal early-pregnancy glycemic index		
	Total group	Women with BMI<25 kg/m^2^	Women with BMI≥25 kg/m^2^
	Difference in BMI SDS (95% CI)		
	*n=2483*	*n=1920*	*n=563*
Basic model^a^	0.05 (0.01; 0.09)**	0.02 (−0.02; 0.06)	0.10 (0.01; 0.19)*
Confounder model^b^	0.02 (−0.01; 0.06)	0.00 (−0.04; 0.04)	0.10 (0.01; 0.18)*
Birth model^c^	0.02 (−0.02; 0.06)	0.00 (−0.04; 0.04)	0.10 (0.01; 0.18)*
Child model^d^	0.02 (−0.02; 0.05)	-0.01 (−0.05; 0.03)	0.11 (0.02; 0.19)*
Maternal diet model^e^	0.01 (−0.03; 0.05)	-0.03 (−0.07; 0.02)	0.11 (0.02; 0.21)*
	Difference in total fat mass index SDS (95% CI)		
	*n=2455*	*n=l898*	*n=557*
Basic model^a^	0.07 (0.03; 0.11)**	0.03 (−0.01; 0.07)	0.15 (0.06; 0.24)**
Confounder model^b^	0.04 (0.00; 0.07)	0.01 (−0.03; 0.05)	0.13 (0.05; 0.22)**
Birth model^c^	0.04 (0.00; 0.07)	0.01 (−0.03; 0.05)	0.13 (0.05; 0.22)**
Child model^d^	0.03 (−0.01; 0.07)	0.00 (−0.04; 0.04)	0.14 (0.05; 0.22)**
Maternal diet model^e^	0.01 (−0.03; 0.05)	-0.03 (−0.07; 0.02)	0.13 (0.03; 0.23)*
	Difference in android/gynoid ratio SDS (95% CI)		
	*n=2458*	*n=1901*	*n*=*557*
Basic model^a^	0.04 (0.00; 0.08)*	0.01 (−0.03; 0.05)	0.11 (0.00; 0.21)
Confounder model^b^	0.01 (−0.03; 0.05)	-0.01 (−0.06; 0.03)	0.09 (−0.01; 0.19)
Birth model^c^	0.01 (−0.03; 0.05)	-0.01 (−0.06; 0.03)	0.09 (−0.01; 0.19)
Child model^d^	0.01 (−0.04; 0.04)	-0.02 (−0.07; 0.02)	0.09 (−0.01; 0.19)
Maternal diet model^e^	-0.02 (−0.07; 0.02)	-0.06 (−0.11; −0.01)	0.09 (−0.03; 0.20)
	Difference visceral fat mass index SDS (95% CI)		
	*n=1246*	*n=956*	*n=290*
Basic model^a^	0.08 (0.02; 0.13)**	0.02 (−0.05; 0.08)	0.23 (0.10; 0.36)**
Confounder model^b^	0.04 (−0.01; 0.10)	0.00 (−0.07; 0.06)	0.19 (0.07; 0.32)**
Birth model^c^	0.04 (−0.01; 0.10)	0.00 (−0.07; 0.06)	0.19 (0.07; 0.32)**
Child model^d^	0.04 (−0.02; 0.09)	-0.01 (−0.07; 0.05)	0.19 (0.07; 0.32)**
Maternal diet model^e^	0.02 (−0.04; 0.08)	-0.02 (−0.10; 0.05)	0.17 (0.03; 0.31)**
	Difference in liver fat fraction SDS (95% CI)		
	*n=1395*	*n=1074*	*n=32l*
Basic model^a^	0.00 (−0.05; 0.06)	0.00 (−0.06; 0.05)	0.00 (−0.14; 0.13)
Confounder model^b^	-0.03 (−0.08; 0.03)	-0.01 (−0.07; 0.05)	-0.05 (−0.18; 0.08)
Birth model^c^	-0.02 (−0.08; 0.03)	-0.01 (−0.07; 0.05)	-0.05 (−0.18; 0.08)
Child model^d^	-0.03 (−0.08; 0.03)	-0.02 (−0.08; 0.04)	-0.05 (−0.19; 0.08)
Maternal diet model^e^	-0.06 (−0.12; 0.00)	-0.05 (−0.12; 0.02)	-0.07 (−0.22; 0.08)

*P < 0.05 **P < 0.01. SDS: standard deviation scores. Values represent regression coefficients (95% confidence interval) from linear regression models that reflect differences in standard deviation score of childhood adiposity outcomes per SDS increase in maternal early-pregnancy dietary glycemic index. One SDS maternal early-pregnancy dietary glycemic index corresponds to an increase of glycemic index of 3.5. P-values for interaction terms maternal prepregnancy BMI*maternal dietary glycemic index for each individual childhood adiposity outcome were 0.11 for BMI, 0.00 for total fat mass index, 0.08 for android/gynoid fat mass ratio, 0.00 for visceral fat mass index and 0.89 for liver fat fraction.

a Basic models were adjusted for gestational age at intake, fetal sex and child’s age at follow up.

b Confounder models were the basic models additionally adjusted for maternal age, maternal educational level, maternal prepregnancy BMI, smoking during pregnancy, vomiting during early-pregnancy, daily total energy intake.

c Birth models were the confounder models additionally adjusted for gestational-age-and-sex adjusted birth weight.

d Child models were the birth models, additionally adjusted for infant breastfeeding, introduction of solid foods and average television watching time.

e Maternal diet models were the confounder models additionally adjusted for gestational weight gain and maternal fiber, fat and protein intake.

**Table 3:** Associations of maternal early-pregnancy dietary glycemic index with risk of childhood overweight.

	Odds Ratio for risk of childhood overweight per SDS increase in maternal early-pregnancy glycemic index		
	Total group	Women with BMI<25 kg/m^2^	Women with BMI≥25 kg/m^2^
Basic model^a^	1.12 (0.99; 1.26)	1.00 (0.85; 1.19)	1.21 (0.99; 1.48)
Confounder model^b^	1.05 (0.92; 1.20)	0.94 (0.79; 1.12)	1.20 (0.97; 1.48)
Birth model^c^	1.06 (0.92; 1.21)	0.94 (0.79; 1.12)	1.21 (0.98; 1.49)
Child model^d^	1.04 (0.91; 1.20)	0.92 (0.76; 1.10)	1.24 (0.99; 1.54)
Maternal diet model^e^	0.98 (0.85; 1.14)	0.84 (0.68; 1.03)	1.19 (0.94; 1.49)

SDS: standard deviation scores. Values represent odds ratios (95% confidence interval) from logistic regression models that reflect differences in standard deviation score of risk of childhood overweight per SDS in maternal early-pregnancy dietary glycemic index. One SDS maternal early-pregnancy dietary glycemic index corresponds to an increase of glycemic index of 3.5. p-value for interaction term maternal prepregnancy weight status*maternal dietary glycemic index was 0.19.

a Basic models were adjusted for gestational age at intake, fetal sex and child’s age at follow up.

b Confounder models were the basic models additionally adjusted for maternal age, maternal educational level, maternal prepregnancy BMI, smoking during pregnancy, vomiting during early-pregnancy, daily total energy intake.

c Birth models were the confounder models additionally adjusted for gestational-age-and-sex adjusted birth weight.

d Child models were the birth models, additionally adjusted for infant breastfeeding, introduction of solid foods and average television watching time.

e Maternal diet models were the confounder models additionally adjusted for gestational weight gain and maternal fiber, fat and protein intake.
