# Supplementary material for: Associations of maternal early-pregnancy dietary glycemic index with childhood general, abdominal and ectopic fat accumulation
Source: Clin Nutr. Author manuscript; Available in PMC 2022 Oct 27. (PMC7613756; doi:10.1016/j.clnu.2021.02.046)
Supplement: Supplementary data [file EMS156031-supplement-Supplementary_data.docx]

**Supplementary material**

Associations of maternal early-pregnancy dietary glycemic index with childhood general, abdominal and ectopic fat accumulation

*Running title: Maternal dietary glycemic index and childhood adiposity*

Rama J. Wahab MD^1,2^, Vincent W.V. Jaddoe MD PhD^1,2^, Romy Gaillard MD PhD^1,2^

1. The Generation R Study Group, Erasmus MC, University Medical Center, Rotterdam, the Netherlands.
2. Department of Pediatrics, Sophia’s Children’s Hospital, Erasmus MC, University Medical Center, Rotterdam, the Netherlands.

**Content**
Methods S1 Log-log regression analyses

Methods S2 Directed Acyclic Graph analysis

Figure S1 Flow chart of the study participants

Table S1 Non-response analysis for Dutch women with information on dietary glycemic index available and singleton livebirths and their offspring with and without participation in follow up measurements at 10 years

Table S2 Associations of maternal early-pregnancy dietary glycemic index in quartiles with childhood general, abdominal and ectopic fat accumulation

Table S3 Associations of maternal early-pregnancy dietary glycemic load with childhood general, abdominal and ectopic fat accumulation

Table S4 Associations of maternal early-pregnancy dietary glycemic load with risk of childhood overweight

Table S5 Associations of maternal early-pregnancy dietary glycemic index with childhood general, abdominal and ectopic fat accumulation after adjustment for maternal DASH diet score

Table S6 Associations of maternal early-pregnancy dietary glycemic index with childhood general, abdominal and ectopic fat accumulation among women without gestational diabetes

**Methods S1**. **Log-log regression analyses**

For our fat measures, we created index variables, which were made independent of height. We did this by dividing our fat measurements by the optimal adjustment for height. The optimal adjustment was determined using log-log regression analyses(1). Total fat mass, visceral fat mass and height were log-transformed using natural logs. We performed linear regression analyses with log-fat measures as the dependent variable and log- height as the independent variable. The regression slope corresponds with the power by which height should be raised. This resulted in the following index values of the fat measures: total fat mass divided by height^4^ and visceral fat mass divided by height^3^.

1. Wells JC, Cole TJ, steam As: Adjustment of fat-free mass and fat mass for height in children aged 8 y. Int J Obes Relat Metab Disord 2002;26:947-952

**Methods S2. Directed Acyclic Graph analysis**

**
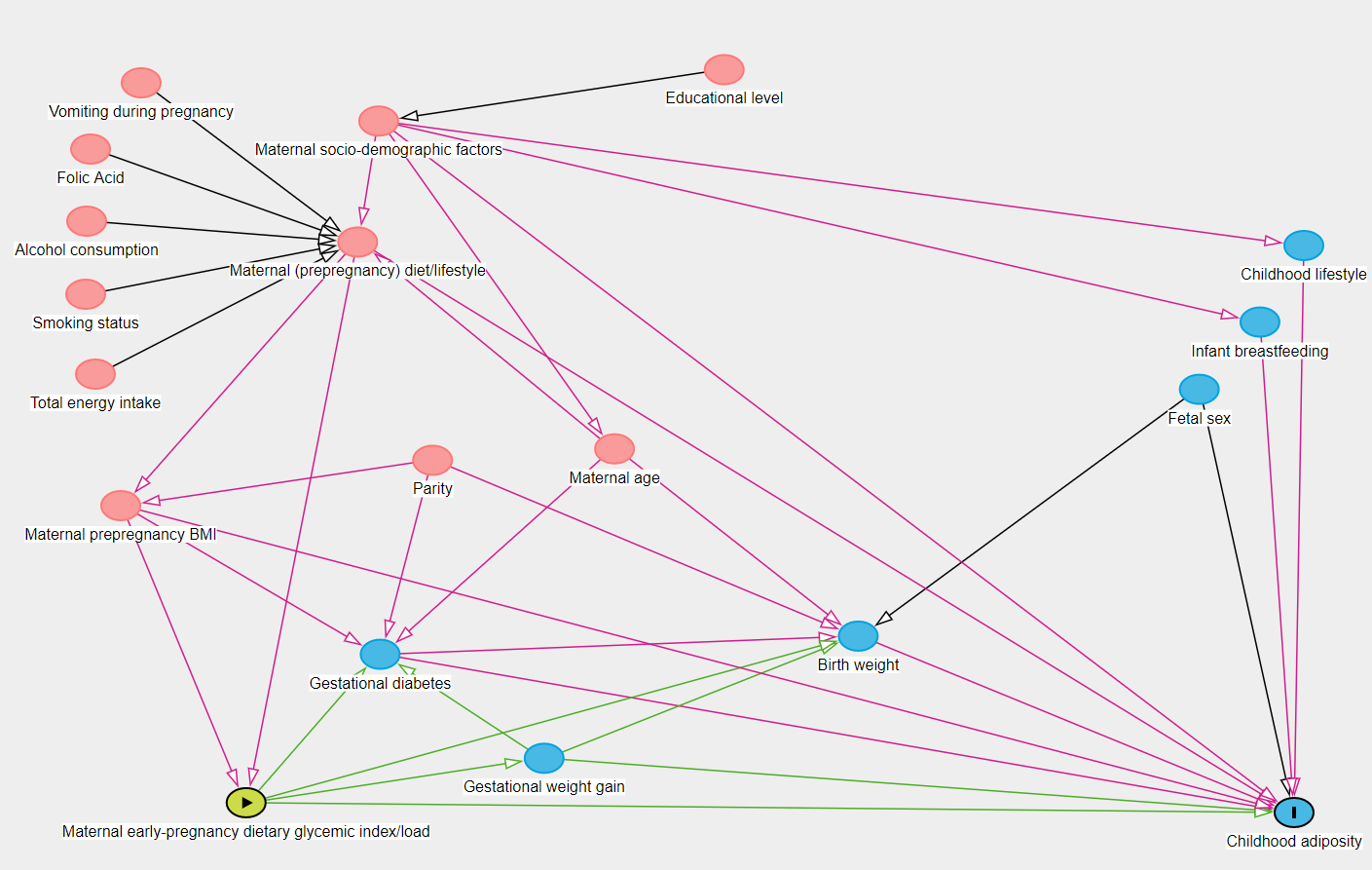
**

**Figure S1. Flow chart of the study participants**

**n= 4,096**

Pregnant women of Dutch ethnicity enrolled during pregnancy

**n= 3,558**

Mothers with information available on dietary intake

**n=2,488**

**Population for analysis:**

Singleton live-births with information on maternal early pregnancy dietary glycemic index and at least one childhood adiposity outcome

BMI n= 2,483

Total fat mass index n=2,458

Android/gynoid fat mass ratio n=2,458

Visceral fat mass index n=1,246

Liver fat fraction n=1,397

**n= 87**

**Excluded:**

Multiple pregnancy n= 53

Induced abortion n= 8

Intrauterine fetal death n= 16

Loss to follow up n= 3

Pregestational diabetes n= 7

**n= 538**

**Excluded:**

No information or non-reliable information available on dietary intake in early-pregnancy

**n= 3,471**

Mothers with information available on dietary intake and singleton live births

**n= 983**

**Excluded:**

Children without follow up measurement at 10 years

**Table S1. Non-response analysis for Dutch women with information on dietary glycemic index available and singleton livebirths and their offspring with and without participation in follow up measurements at 10 years**

|  | Participation in offspring follow up measurements at 10 years (n=2,488) | No participation in offspring follow up measurements at 10 years  (n=983) | p-value |
| --- | --- | --- | --- |
| Maternal characteristics |  |  |  |
| Maternal age at enrolment, mean (SD), years | 31.8 (4.1) | 30.3 (4.8) | 0.00 |
| Gestational age at enrolment, median (95% range), weeks | 13.4 (9.9; 22.6) | 13.4 (9.7; 24.0) | 0.85 |
| Parity, n nulliparous (%) | 1,549 (62.3) | 532 (54.0) | 0.00 |
| Pre-pregnancy BMI, median (95% range) | 22.3 (18.4; 33.3) | 22.2 (18.0; 34.1) | 0.72 |
| Gestational weight gain, mean (SD), kg/week | 0.35 (0.14) | 0.36 (0.15) | 0.04 |
| Education, n high (%) | 1,566 (62.9) | 461 (47.2) | 0.00 |
| Glycemic index, mean (SD) | 57.7 (3.5) | 58.0 (3.2) | 0.40 |
| Glycemic load, mean (SD) | 154.0 (46.1) | 156.1 (48.4) | 0.24 |
| Low glycemic index, n (%) | 522 (20.9) | 183 (26.0) | 0.10 |
| Carbohydrate intake, mean (SD), g/d | 266 (74) | 269 (78) | 0.20 |
| Protein intake, mean (SD), g/d | 79 (19) | 78 (20) | 0.10 |
| Fat intake, mean (SD), g/d | 87 (24) | 86 (25) | 0.05 |
| Fiber intake, mean (SD), g/d | 24 (7) | 23 (7) | 0.23 |
| Total energy intake, mean (SD), kcal/d | 2145 (500) | 2,145 (538) | 0.05 |
| Folic acid supplement use, n yes (%) | 1,865 (75.0) | 672 (83.1) | 0.00 |
| Alcohol use during pregnancy, n yes (%) | 1,577 (63.4) | 540 (25.5) | 0.00 |
| Smoking during pregnancy, n yes (%) | 510 (20.5) | 324 (34.9) | 0.00 |
| Vomiting during early-pregnancy, n yes (%) | 93 (3.7) | 52 (5.7) | 0.05 |
| Gestational diabetes, n (%) | 20 (0.8) | 12 (1.3) | 0.24 |
| Birth/infant characteristics |  |  |  |
| Sex, n female (%) | 1,255 (50.4) | 468 (47.3) | 0.09 |
| Gestational age at birth, median (95% range), weeks | 40.3 (36.0; 42.4) | 40.1 (35.3; 42.3) | 0.00 |
| Birthweight, mean (SD), g | 3498 (537) | 3,465 (596) | 0.00 |
| Ever breastfed, yes (%) | 2006 (80.6) | 587 (85.7) | 0.00 |
| Introduction of solid foods before 6 months, yes (%) | 1633 (65.6) | 444 (87.2) | 0.75 |

BMI: body mass index. P-values were obtained by independent t-test or Mann-Whitney U-test for continuous variables and chi-square tests for categorical variables.

**Table S2. Associations of maternal early-pregnancy dietary glycemic index in quartiles with childhood general, abdominal and ectopic fat accumulation**

|  | Effect estimates for childhood outcome for maternal early-pregnancy glycemic index in quartiles | | |
| --- | --- | --- | --- |
|  | Total group | Women with BMI<25 kg/m^2^ | Women with BMI≥25kg/m^2^ |
|  | **Difference in BMI SDS (95% CI)** | | |
|  | *2483* | *1920* | *563* |
| First quartile (n=621) | *Reference* | *Reference* | *Reference* |
| Second quartile (n=621) | 0.08 (-0.02; 0.18) | -0.01 (-0.12; 0.10) | 0.41 (0.16; 0.66)* |
| Third quartile (n=619) | 0.08 (-0.03; 0.18) | 0.05 (-0.06; 0.16) | 0.19 (-0.07; 0.44) |
| Fourth quartile (n=622) | 0.06 (-0.05; 0.16) | -0.02 (-0.13; 0.10) | 0.31 (0.06; 0.57)* |
|  | **Difference in total fat mass index**  **SDS (95% CI)** | | |
|  | *2455* | *1898* | *557* |
| First quartile (n=611) | *Reference* | *Reference* | *Reference* |
| Second quartile (n=618) | 0.13 (0.03; 0.24)* | 0.07 (-0.05; 0.18) | 0.42 (0.17; 0.67)* |
| Third quartile (n=611) | 0.07 (-0.04; 0.17) | 0.04 (-0.08; 0.15) | 0.22 (-0.04; 0.47) |
| Fourth quartile (n=615) | 0.10 (-0.01; 0.21) | 0.02 (-0.10; 0.14) | 0.40 (0.15; 0.66)* |
|  | **Difference in android/gynoid ratio**  **SDS (95% CI)** | | |
|  | *2458* | *1901* | *557* |
| First quartile (n=611) | *Reference* | *Reference* | *Reference* |
| Second quartile (n=619) | 0.07 (-0.04; 0.19) | 0.02 (-0.10; 0.14) | 0.30 (0.02; 0.58)* |
| Third quartile (n=613) | 0.00 (-0.11; 0.12) | -0.04 (-0.16; 0.08) | 0.17 (-0.12; 0.45) |
| Fourth quartile (n=615) | 0.01 (-0.11; 0.12) | -0.05 (-0.17; 0.08) | 0.22 (-0.07; 0.52) |
|  | **Difference visceral fat mass index**  **SDS (95% CI)** | | |
|  | *1246* | *956* | *290* |
| First quartile (n=307) | *Reference* | *Reference* | *Reference* |
| Second quartile (n=313) | 0.18 (0.02; 0.33)* | 0.09 (-0.09; 0.26) | 0.56 (0.19; 0.92)* |
| Third quartile (n=321) | 0.09 (-0.7; 0.25) | 0.05 (-0.12; 0.22) | 0.32 (-0.04; 0.69) |
| Fourth quartile (n=305) | 0.10 (-0.07; 0.26) | -0.06 (-0.24; 0.12) | 0.65 (0.28; 1.02)* |
|  | **Difference in liver fat fraction SDS (95% CI)** | | |
|  | *1395* | *1074* | *321* |
| First quartile (n=351) | *Reference* | *Reference* | *Reference* |
| Second quartile (n=347) | -0.02 (-0.17; 0.13) | -0.01 (-0.17; 0.16) | -0.05 (-0.43; 0.34) |
| Third quartile (n=355) | -0.09 (-0.24; 0.07) | -0.06 (-0.23; 0.10) | -0.15 (-0.54; 0.23) |
| Fourth quartile (n=344) | -0.05 (-0.21; 0.11) | 0.01 (-0.16; 0.18) | -0.23 (-0.62; 0.16) |

*P<0.05 SDS: standard deviation scores

Values represent regression coefficients (95% confidence interval) from linear regression models that reflect differences in standard deviation score of childhood adiposity outcomes for the upper three maternal dietary glycemic index quartiles as compared to the lowest quartile.

^a^Basic models were adjusted for gestational age at intake, fetal sex and child’s age at follow up

^b^Confounder models were the basic models additionally adjusted for maternal age, maternal educational level, maternal prepregnancy BMI, smoking during pregnancy, vomiting during early-pregnancy, daily total energy intake

^c^Birth models were the confounder models additionally adjusted for gestational-age-and-sex adjusted birth weight

^d^Child models were the birth models, additionally adjusted for infant breastfeeding, introduction of solid foods and average television watching time

^e^Maternal diet models were the confounder models additionally adjusted for gestational weight gain and maternal fiber, fat and protein intake

**Table S3. Associations of maternal early-pregnancy dietary glycemic load with childhood general, abdominal and ectopic fat accumulation**

|  | Effect estimates for childhood outcome per SDS increase in maternal early-pregnancy glycemic load | | |
| --- | --- | --- | --- |
|  | Total group | Women with BMI<25 kg/m^2^ | Women with BMI≥25kg/m^2^ |
|  | **Difference in BMI SDS (95% CI)** | | |
|  | *2483* | *1920* | *563* |
| Basic model^a^ | -0.01 (-0.04; 0.03) | 0.00 (-0.04; 0.04) | 0.02 (-0.07; 0.10) |
| Confounder model^b^ | 0.09 (0.02; 0.15) | 0.07 (0.00; 0.15) | 0.12 (-0.04; 0.28) |
| Birth model^c^ | 0.08 (0.02; 0.15) | 0.07 (0.00; 0.14) | 0.13 (-0.03; 0.28) |
| Child model^d^ | 0.08 (0.01; 0.15) | 0.06 (-0.01; 0.13) | 0.15 (-0.01; 0.30) |
| Maternal diet model^e^ | 0.05 (-0.14; 0.24) | -0.09 (-0.30; 0.12) | 0.51 (0.03; 0.98) |
|  | **Difference in total fat mass index**  **SDS (95% CI)** | | |
|  | 2455 | 1898 | 557 |
| Basic model^a^ | -0.01 (-0.05; 0.03) | -0.01 (-0.05; 0.03) | 0.04 (-0.04; 0.13) |
| Confounder model^b^ | 0.08 (0.01; 0.15)* | 0.06 (-0.02; 0.13) | 0.16 (0.00; 0.32) |
| Birth model^c^ | 0.08 (0.01; 0.15)* | 0.06 (-0.02; 0.13) | 0.15 (0.00; 0.33) |
| Child model^d^ | 0.07 (0.01; 0.14)* | 0.05 (-0.03; 0.12) | 0.17 (0.01; 0.34)* |
| Maternal diet model^e^ | 0.07 (-0.12; 0.26)* | -0.08 (-0.30; 0.13) | 0.61 (0.13; 1.08)* |
|  | **Difference in android/gynoid ratio**  **SDS (95% CI)** | | |
|  | 2458 | 1901 | 557 |
| Basic model^a^ | 0.00 (-0.04; 0.04) | -0.01 (-0.05; 0.04) | 0.03 (-0.07; 0.12) |
| Confounder model^b^ | 0.04 (-0.04; 0.11) | 0.02 (-0.06; 0.10) | 0.09 (-0.09; 0.28) |
| Birth model^c^ | 0.03 (-0.04; 0.11) | 0.02 (-0.06; 0.11) | 0.09 (-0.09; 0.28) |
| Child model^d^ | 0.03 (-0.04; 0.11) | 0.01 (-0.07; 0.10) | 0.10 (-0.09; 0.28) |
| Maternal diet model^e^ | -0.09 (-0.30; 0.11) | -0.22 (-0.46; 0.01) | 0.35 (0.19; 0.90) |
|  | **Difference visceral fat mass index**  **SDS (95% CI)** | | |
|  | 1246 | 956 | 290 |
| Basic model^a^ | -0.03 (-0.09; 0.02) | -0.03 (-0.09; 0.03) | -0.01 (-0.13; 0.12) |
| Confounder model^b^ | 0.10 (-0.01; 0.20) | 0.04 (-0.07; 0.16) | 0.30 (0.06; 0.54)* |
| Birth model^c^ | 0.09 (-0.01; 0.20) | 0.04 (-0.07; 0.16) | 0.30 (0.05; 0.54)* |
| Child model^d^ | 0.08 (-0.03; 0.18) | 0.02 (-0.09; 0.14) | 0.30 (0.05; 0.55)* |
| Maternal diet model^e^ | 0.01 (-0.27; 0.30) | -0.15 (-0.47; 0.17) | 0.67 (-0.01; 1.34) |
|  | **Difference in liver fat fraction SDS (95% CI)** | | |
|  | 1395 | 1074 | 321 |
| Basic model^a^ | 0.00 (-0.06; 0.05) | -0.01 (-0.07; 0.05) | 0.07 (-0.04; 0.19) |
| Confounder model^b^ | 0.04 (-0.06; 0.14) | 0.05 (-0.06; 0.16) | 0.02 (-0.24; 0.27) |
| Birth model^c^ | 0.03 (-0.07; 0.14) | 0.05 (-0.06; 0.16) | 0.01 (-0.25; 0.26) |
| Child model^d^ | 0.03 (-0.08; 0.13) | 0.04 (-0.07; 0.15) | -0.01 (-0.26; 0.25) |
| Maternal diet model^e^ | 0.32 (-0.59; -0.04) | -0.22 (-0.52; 0.08) | -0.67 (-1.37; 0.03) |

*P<0.05 SDS: standard deviation scores

Values represent regression coefficients (95% confidence interval) from linear regression models that reflect differences in standard deviation score of childhood adiposity outcomes per SDS increase in maternal early-pregnancy dietary glycemic load.

^a^Basic models were adjusted for gestational age at intake, fetal sex and child’s age at follow up

^b^Confounder models were the basic models additionally adjusted for maternal age, maternal educational level, maternal prepregnancy BMI, smoking during pregnancy, vomiting during early-pregnancy, daily total energy intake

^c^Birth models were the confounder models additionally adjusted for gestational-age-and-sex adjusted birth weight

^d^Child models were the birth models, additionally adjusted for infant breastfeeding, introduction of solid foods and average television watching time

^e^Maternal diet models were the confounder models additionally adjusted for gestational weight gain and maternal fiber, fat and protein intake

**Table S4. Associations of maternal early-pregnancy dietary glycemic load with risk of childhood overweight**

|  | Effect estimates for risk of childhood overweight per SDS increase in maternal early-pregnancy glycemic load | | |
| --- | --- | --- | --- |
|  | Total group | Women with BMI<25 kg/m^2^ | Women with BMI≥25kg/m^2^ |
| Basic model^a^ | 1.03 (0.91; 1.17) | 0.98 (0.82; 1.17) | 1.17 (0.97; 1.41) |
| Confounder model^b^ | 1.25 (0.98; 1.60) | 1.09 (0.78; 1.52) | 1.47 (1.00; 2.17) |
| Birth model^c^ | 1.25 (0.98; 1.60) | 1.08 (0.77; 1.51) | 1.49 (1.01; 2.20)* |
| Child model^d^ | 1.24 (1.10; 1.86)* | 1.05 (0.75; 1.47) | 1.56 (1.05; 2.32)* |
| Maternal diet model^e^ | 1.05 (0.53; 2.10) | 0.60 (0.23; 1.54) | 2.01 (0.67; 6.05) |

*P<0.05

SDS: standard deviation scores

Values represent odds ratios (95% confidence interval) from logistic regression models that reflect differences in standard deviation score of childhood risk of overweight per SDS in maternal early-pregnancy dietary glycemic load.

^a^Basic models were adjusted for gestational age at intake, fetal sex and child’s age at follow up

^b^Confounder models were the basic models additionally adjusted for maternal age, maternal educational level, maternal prepregnancy BMI, smoking during pregnancy, vomiting during early-pregnancy, daily total energy intake

^c^Birth models were the confounder models additionally adjusted for gestational-age-and-sex adjusted birth weight

^d^Child models were the birth models, additionally adjusted for infant breastfeeding, introduction of solid foods and average television watching time

^e^Maternal diet models were the confounder models additionally adjusted for gestational weight gain and maternal fiber, fat and protein intake

**Table S5. Associations of maternal early-pregnancy dietary glycemic index with childhood general, abdominal and ectopic fat accumulation after adjustment for maternal DASH diet score**

|  | Effect estimates for childhood outcome per SDS increase in maternal early-pregnancy glycemic load | | |
| --- | --- | --- | --- |
|  | Total group | Women with BMI<25 kg/m^2^ | Women with BMI≥25kg/m^2^ |
|  | **Difference in BMI SDS (95% CI)** | | |
|  | *2483* | *1920* | *563* |
| Confounder model additionally adjusted for maternal DASH diet score^a^ | 0.04 (0.00; 0.08) | 0.01 (-0.04; 0.05) | 0.12 (0.02; 0.21)* |
|  | **Difference in total fat mass index**  **SDS (95% CI)** | | |
|  | *2455* | *1898* | *557* |
| Confounder model additionally adjusted for maternal DASH diet score^a^ | 0.03 (-0.01; 0.01) | -0.01 (-0.05; 0.04) | 0.13 (0.03; 0.23)* |
|  | **Difference in android/gynoid ratio**  **SDS (95% CI)** | | |
|  | *2458* | *1901* | *557* |
| Confounder model additionally adjusted for maternal DASH diet score^a^ | -0.01 (-0.05; 0.04) | -0.04 (-0.09; 0.01) | 0.08 (-0.03; 0.19) |
|  | **Difference visceral fat mass index**  **SDS (95% CI)** | | |
|  | *1246* | *956* | *290* |
| Confounder model additionally adjusted for maternal DASH diet score^a^ | 0.04 (-0.02; 0.10) | 0.00 (-0.08; 0.07) | 0.17 (0.03; 0.31)* |
|  | **Difference in liver fat fraction SDS (95% CI)** | | |
|  | *1395* | *1074* | *321* |
| Confounder model additionally adjusted for maternal DASH diet score^a^ | -0.03 (-0.09; 0.03) | -0.02 (-0.09; 0.05) | -0.05 (-0.19; 0.09) |

*P<0.05 SDS: standard deviation scores. DASH: Dietary Approaches to Stop Hypertension

Values represent regression coefficients (95% confidence interval) from linear regression models that reflect differences in standard deviation score of childhood adiposity outcomes per SDS increase in maternal early-pregnancy dietary glycemic index adjusted according to confounder models with additional adjustment for maternal DASH diet score

^a^Confounder models were adjusted for gestational age at intake, fetal sex and child’s age at follow up, maternal age, maternal educational level, maternal prepregnancy BMI, smoking during pregnancy, vomiting during early-pregnancy, daily total energy intake

**Table S6. Associations of maternal early-pregnancy dietary glycemic index with childhood general, abdominal and ectopic fat accumulation among women without gestational diabetes**

|  | Effect estimates for childhood outcome per SDS increase in maternal early-pregnancy glycemic index excluding women with gestational diabetes | | |
| --- | --- | --- | --- |
|  | Total group | Women with BMI<25 kg/m^2^ | Women with BMI≥25kg/m^2^ |
|  | **Difference in BMI SDS (95% CI)** | | |
|  | *n=2463* | *n=1911* | *n=552* |
| Confounder model^a^ | 0.02 (-0.02; 0.06) | 0.00 (-0.04; 0.05) | 0.08 (-0.02; 0.17) |
|  | **Difference in total fat mass index**  **SDS (95% CI)** | | |
|  | *n=2435* | *n=1889* | *n=546* |
| Confounder model^a^ | 0.03 (-0.01; 0.07) | 0.00 (-0.04; 0.05) | 0.13 (0.04; 0.23)** |
|  | **Difference in android/gynoid ratio**  **SDS (95% CI)** | | |
|  | *n=2438* | *n=1892* | *n=546* |
| Confounder model^a^ | 0.01 (-0.03; 0.05) | -0.01 (-0.06; 0.03) | 0.08 (-0.03; 0.18) |
|  | **Difference visceral fat mass index**  **SDS (95% CI)** | | |
|  | *n=1241* | *n=954* | *n=287* |
| Confounder model^a^ | 0.06 (0.00; 0.12) | 0.01 (-0.06; 0.08) | 0.22 (0.09; 0.35)** |
|  | **Difference in liver fat fraction SDS (95% CI)** | | |
|  | *n=1392* | *n=1074* | *n=318* |
| Confounder model^a^ | -0.03 (-0.08; 0.03) | 0.00 (-0.07; 0.07) | -0.03 (-0.18; 0.11) |

*P<0.05 **P<0.01

^a^Confounder models were the basic models additionally adjusted for maternal age, maternal educational level, maternal prepregnancy BMI, smoking during pregnancy, vomiting during early-pregnancy, daily total energy intake
